# Wild *Trypanosoma cruzi* I genetic diversity in Brazil suggests admixture and disturbance in parasite populations from the Atlantic Forest region

**DOI:** 10.1186/1756-3305-7-263

**Published:** 2014-06-05

**Authors:** Valdirene S Lima, Ana M Jansen, Louisa A Messenger, Michael A Miles, Martin S Llewellyn

**Affiliations:** 1 Fundação Oswaldo Cruz, Rio de Janeiro, Brazil; 2 Faculty of Infectious and Tropical Diseases, London School of Hygiene and Tropical Medicine, London, UK; 3 Molecular Ecology and Fisheries Genetics Laboratory, School of Biological Sciences, Bangor University, Gwynedd LL57 2UW, UK

## Abstract

**Background:**

*Trypanosoma cruzi* (Kinetoplastida, Trypanosomatidae) infection is an ancient and widespread zoonosis distributed throughout the Americas. Ecologically, Brazil comprises several distinct biomes: Amazonia, Cerrado, Caatinga, Pantanal and the Atlantic Forest. Sylvatic *T. cruzi* transmission is known to occur throughout these biomes, with multiple hosts and vectors involved. Parasite species-level genetic diversity can be a useful marker for ecosystem health. Our aims were to: investigate sylvatic *T. cruzi* genetic diversity across different biomes, detect instances of genetic exchange, and explore the possible impact of ecological disturbance on parasite diversity at an intra-species level.

**Methods:**

We characterised 107 isolates of *T. cruzi* I (TcI; discrete typing unit, DTU I) from different major Brazilian biomes with twenty-seven nuclear microsatellite loci. A representative subset of biologically cloned isolates was further characterised using ten mitochondrial gene loci. We compared these data generated from Brazilian TcI isolates from around America.

**Results:**

Genetic diversity was remarkably high, including one divergent cluster that branched outside the known genetic diversity of TcI in the Americas. We detected evidence for mitochondrial introgression and genetic exchange between the eastern Amazon and Caatinga. Finally, we found strong signatures of admixture among isolates from the Atlantic Forest region by comparison to parasites from other study sites.

**Conclusions:**

Atlantic Forest sylvatic TcI populations are highly fragmented and admixed by comparison to others around Brazil. We speculate on: the possible causes of Atlantic Forest admixture; the role of *T. cruzi* as a sentinel for ecosystem health, and the impact disrupted sylvatic transmission cycles might have on accurate source attribution in oral outbreaks.

## Background

*Trypanosoma cruzi* (Kinetoplastida, Trypanosomatidae) infection is an ancient and widespread zoonosis distributed throughout the Americas south of 33′ latitude, where it infects approximately 8 million people [1,2]. *T. cruzi* is eclectic in terms of its mammalian hosts and haematophagous triatomine vectors. Several hundred species of mammal and many of the 140 extant triatomine species maintain transmission of *T. cruzi* in wild (sylvatic) transmission cycles [2-4]. Transmission to the host occurs usually via contamination of the mucosae or abraded skin with infected vector faeces. Oral transmission to humans via contaminated foods, especially fruit juices and sugar cane, is increasingly reported, and suspected to occur widely among sylvatic mammals through opportunistic insectivory of triatomines [5].

*T. cruzi* population genetic diversity is well described at a species level. Six discrete typing units (DTUs) are now accepted by international consensus [6]. Dates for the origin of *T. cruzi* in the Americas range between 5 and 1 MYA (calibrated biogeographically at 100 MYA) [7-9]. Estimates for the MRCA of TcI strains, arguably the most widely dispersed and abundant of all the DTUs, are younger: 1.3-0.2 MYA [7]. Nonetheless, the age of TcI in the Americas has been sufficient to see this genotype expand throughout multiple ecological settings, from Amazonian forests [10] to highland Andean puna [11]. Furthermore, the last 1.3-0.2 MYA in Latin America have seen intense climatic fluctuations, including at least two glaciations [12]. The impact of Pleistocene cycles of warming and cooling on the biomic, ecological and species diversity of Latin America, in particular in Brazil and the Brazilian Amazon, are a matter of long debate [13]. Nonetheless, there is evidence that historical cycles of forest expansion, contraction and fragmentation have impacted on the current ecology of Brazil, including small mammal distribution and diversity [14].

Today the terrestrial ecology of Brazil is summarized by several distinct biomes or ‘ecoregions’ [15]. The largest of these is the Amazon basin to the north, bordered by the dryer Cerrado and seasonally flooded Pantanal to the south. North-eastern Brazil is dominated by the xeric scrubland of the Caatinga. Along the Atlantic coast of Brazil south of Recife, a tropical forest ecosystem, the Atlantic Forest, predominates. The diversity of wild TcI hosts across this ecological mosaic is striking: caviomorph rodents in the Caatinga [16]; lion tamarins in the Atlantic forest [17]; coatis, peccaries and felid carnivores in the Pantanal [18-20]; and multiple species of primates, marsupials and rodents in Amazonia [2]. Some important genera are widespread – especially Didelphid opossums. Human Chagas disease was once widespread in Brazil, especially in central and southern parts of the country [21]. Indeed, Chagas disease has probably been endemic in human populations in Brazil since the earliest human settlements more than 10,000 years ago. It is important not to overlook the impact that humans, an abundant and mobile *T. cruzi* host species, present throughout all Brazilian ecoregions, may have had on contemporary parasite diversity.

Parasite alpha diversity at a species level is recognised as a marker for ecosystem persistence, productivity, organization and resilience [22]. Put simply, those ecosystems in which host organisms are parasitized by an array of different parasite species, fairly evenly distributed among hosts and host species, are considered to be healthy. Furthermore, parasites, with their short lifecycles and rapid mutational turnover with respect to their hosts, can facilitate fine-scale analyses of host population dispersal and differentiation [23]. However, close association between host and parasite species is a prerequisite for the use of parasite genetic diversity to track host populations. Multi-host parasite lineages like TcI are therefore unsuitable for such applications. Nonetheless, there is some evidence that habitat fragmentation impacts on both *T. cruzi* diversity and prevalence of infection [24-26]. Thus, alpha diversity in a multi-host parasite like *T. cruzi* might be a useful proxy for parasite diversity as a whole, and thus for ecosystem health.

Multilocus microsatellite typing (MLMT) is now a widely established means of defining genetic diversity among TcI isolates and clones [27]. Simultaneous analysis of multilocus sequence data from the mitochondrial (maxicircle) genome (mMLST) provides a proven means of detecting genetic exchange among clones [25,28]. Here we undertook a comparison of representative TcI isolates from across the ecological diversity of Brazil, examining the relationship between biomes and diversity within biomes. We found considerable genetic diversity among several populations, and multiple instances of genetic admixture, especially in the Atlantic Forest region. We consider these data, and the potential affect of human-mediated habitat fragmentation on the diversity of wild TcI in Brazil.

## Methods

### Parasite strains and biological cloning

**Figure 1:**
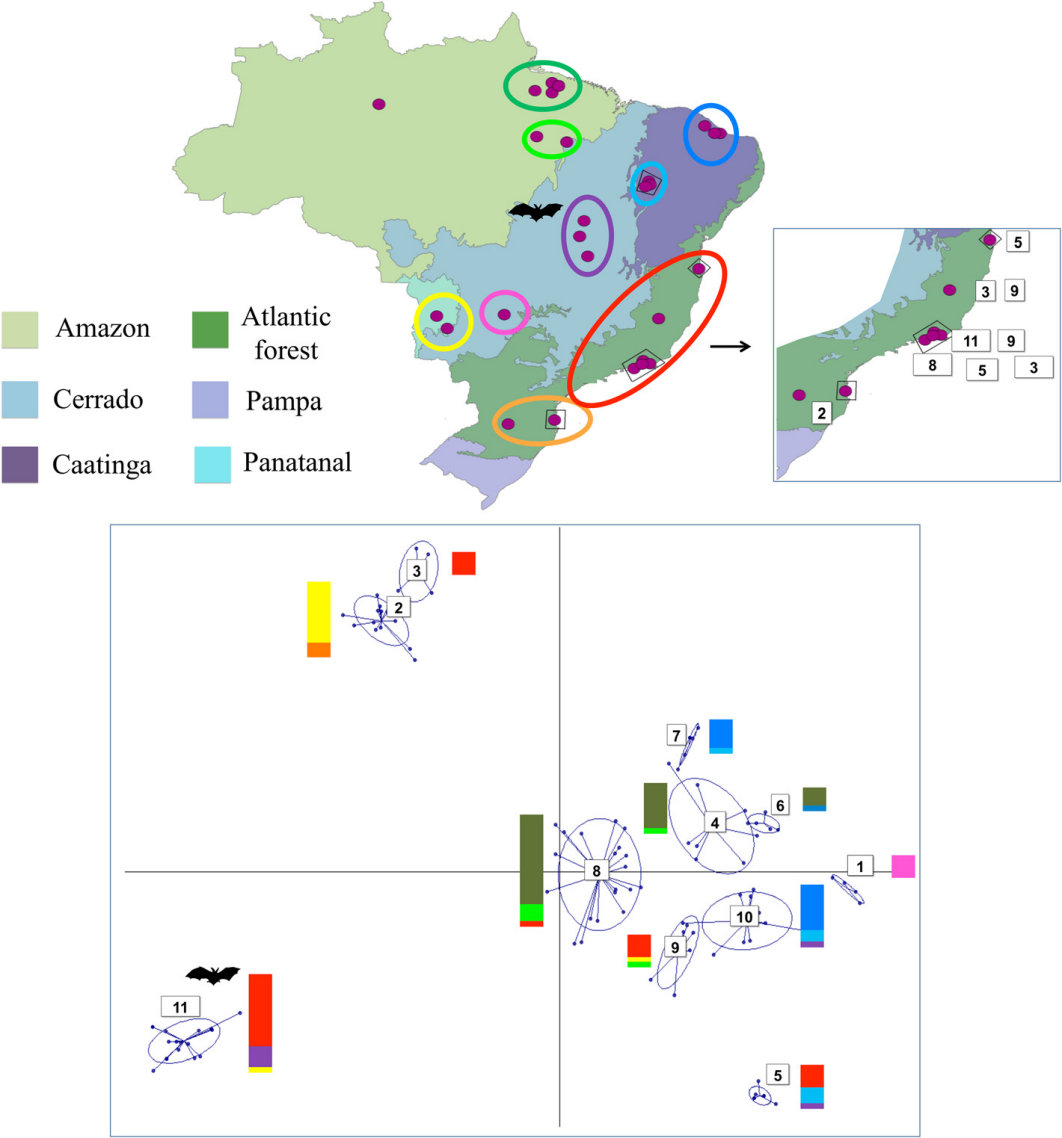
**Composite map and multidimensional scaling plot depicting sample clustering by biome and geography among 107 *Trypanosoma cruzi *** **I isolates.**

One hundred and seven strains, the great majority sampled from mammalian reservoir hosts captured at sylvatic foci throughout Brazil, were assembled for analysis and their genotype confirmed as TcI via sequencing of a short fragment of the glucose-6-phosphate isomerase (GPI) gene [29]. Details of strain origin are given in Additional file 1: Table S1 and geographic distribution in Figure 1. A total of fourteen strains were selected from across all biomes and biologically cloned using the plate cloning technique described by Yeo *et al*. [30].

### Microsatellite analysis

Twenty-seven microsatellite loci, distributed across eight putative chromosomes, were amplified following previously described protocols across 107 strains [27]. A reduced subset of 19 microsatellites was employed to evaluate diversity among a larger panel of 161 samples including the original strains, derived clones and thirty-three previously published multilocus microsatellite profiles [28]. Population genetic diversity parameters were first calculated from sample groupings based on geography and biome for the full 27 locus dataset (Table 1). There were nine such groupings, as identified in Figure 1 and listed in Additional file 1: Table S1. Population-level genetic diversity was assessed first using sample size corrected allelic richness (A_r_) in FSTAT 2.9.3.2 [31]. Secondly, to provide a better measure on intra-population sub-clustering, mean pairwise *D*_AS_ and associated standard deviation was also evaluated per population. *F*_IS_, a measure of the distribution of heterozygosity within and between individuals, was estimated per locus per population in FSTAT 2.9.3.2 [31]. Tests for population specific departures from Hardy Weinberg Equilibrium at specific loci were calculated in ARLEQUIN v3.1 and associated significance levels for p values derived after sequential Bonferroni correction to minimise the likelihood of Type 1 errors [32].

**Table 1:** **Population genetic parameters across nine *Trypanosoma cruzi *** **I populations sampled from five biomes in Brazil**

**Population**	**N**	**A**_**r**_ **± SE**	**D**_**AS**_ **± SD**	**% PL H**_**E**_^**a**^	**% PL Hd**^**b**^	**F**_**IS**_ **± SE**^**c**^
Ceara	14	1.746 ± 0.121	0.290 ± 0.131	0	0	0.020 ± 0.012
Goais	4	1.734 ± 0.101	0.136 ± 0.067	0	0	-0.526 ± 0.032
*PARA*_NORTH_	28	2.134 ± 0.143	0.445 ± 0.082	0	19.2	0.147 ± 0.008
*PARA*_SOUTH_	5	2.027 ± 0.152	0.416 ± 0.053	0	0	0.250 ± 0.019
Pantanal	13	1.698 ± 0.121	0.219 ± 0.197	26.3	5.2	0.068 ± 0.029
Piaui	6	1.930 ± 0.140	0.357 ± 0.188	0	0	0.080 ± 0.023
Atlantic Forest	27	2.010 ± 0.133	0.369 ± 0.199	33.3	33.3	0.077 ± 0.015
Santa Catarina	3	1.412 ± 0.098	0.057 ± 0.020	0	0	-0.740 ± 0.033
Tocantins	7	1.959 ± 0.133	0.362 ± 0.221	14.2	0	0.180 ± 0.025

*N* number of isolates in population.

*A*_*r*_ allelic richness as a mean over loci **±** standard error**,** calculated in FSTAT.

*D*_*AS*_ mean pair-wise inverse allele sharing between samples **±** standard deviation calculated in MICROSAT.

^a^Proportion of loci showing significant excess heterozygosity after a sequential Bonferroni correction. Calculated in ARLEQUIN v3.1.

^b^Proportion of loci showing a significant deficit in heterozygosity after a sequential Bonferroni correction. Calculated in ARLEQUIN v3.1.

^c^Mean FIS over loci **±** standard error, calculated in FSTAT.

**Figure 2:**
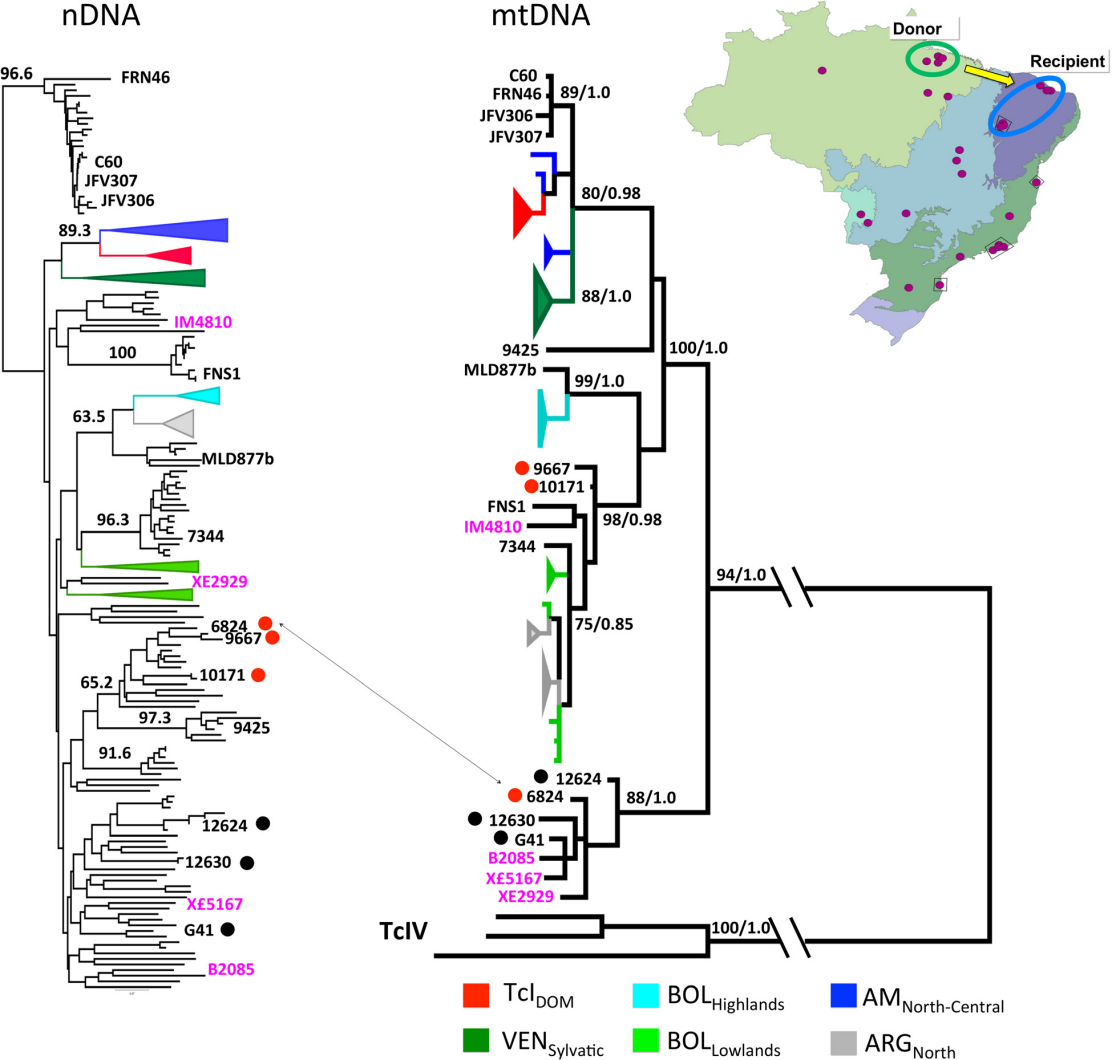
**Comparison of phylogenetic clustering between nuclear and mitochondrial phylogenies among *Trypanasoma cruzi *** **I isolates from Brazil and beyond reveals genetic exchange.**

For the 19 locus dataset, individual level sample clustering was defined via a neighbour-joining tree based on pairwise distances between multilocus genotypes MLGs [evaluated using *D*_AS_ (1 − proportion of shared alleles at all loci/*n*)] calculated in MICROSAT [33] (Figure 2). For the 27 locus dataset we defined genetic composition via a *K*-means clustering algorithm, implemented in adegenet [34], with which the optimal number of populations is defined by reference to the Bayesian Information Criterion. These groupings were subsequently submitted to a discriminant analysis of principal components (DAPC) [35], and the resulting plot is found in Figure 1.

### Maxicircle analysis

Ten maxicircle sequence fragments were amplified and sequenced from fourteen *T. cruzi* clones (see Additional file 1: Table S1 for clone identity) following previously described protocols [28]. Sequence fragments were then concatenated in each sample and aligned against previously published sequences prior to analysis [28]. Phylogenies were inferred using Maximum-Likelihood (ML) implemented in PhyML (4 substitution rate categories) [36]. The best-fit model of nucleotide substitution was selected from 88 models and its significance evaluated according to the Akaike Information Criterion (AIC) in jMODELTEST 1.0 [37]. The best model selected for this dataset was GTR + I + G. Bootstrap support for clade topologies was estimated following the generation of 1000 pseudo-replicate datasets. Bayesian phylogenetic analysis was performed using Mr BAYES v3.1 [38] (settings according to jMODELTEST 1.0). Five independent analyses were run using a random starting tree with three heated chains and one cold chain over 10 million generations with sampling every 10 simulations (25% burn-in).

## Results

Nuclear microsatellite loci demonstrated considerable genetic diversity among the 107 strains studied. For comparative purposes isolates were grouped *a priori* according to both geography and biome of origin (Figure 1). As such nine populations were defined. Sample assignment to these populations is presented in Additional file 1: Table S1 and population genetic parameters associated with them in Table 1. Of primary interest are sample size corrected values for allelic richness (A_r_). A_r_ is highest among *PARA*_NORTH_ and *PARA*_SOUTH_ samples in the Eastern Amazon (A_r_ = 2.027 & 2.134), as well as in the Atlantic Forest (A_r_ = 2.010) and Tocantins, in the Cerrado (A_r_ 1.959). While A_r_ is a useful measure of overall sample size corrected genetic diversity, structured diversity within a population may be overlooked. We thus also calculated mean pairwise allele sharing (D_AS_) between multilocus genotypes (MLGs) in each population – Table 1. The standard deviations associated with mean D_AS_ values are particularly informative. Diverse populations with elevated standard deviations (e.g. Atlantic Forest – 0.369 ± 0.199, Tocantins - 0.362 ± 0.221) are likely to possess intra-population sub-clusters. By contrast genetic diversity is uniformly distributed among samples within populations with low standard deviations about the mean D_AS_ (*PARA*_NORTH_ - 0.445 ± 0.082, *PARA*_SOUTH_ - 0.416 ± 0.053). Observed heterozygosity varied considerably across populations. However, where population sizes (N > 10) are likely to facilitate meaningful interpretation, positive values for *F*_IS_ prevailed, and by inference heterozygous deficit compared to Hardy-Weinberg expectations (Table 1).

Sample clustering based on pair-wise nuclear genetic distances provides insight into the idiosyncratic patterns of genetic diversity noted across populations. As such, considerable admixture is present between multiple populations. This phenomenon is best represented by the composite bars adjacent to the clusters in the multidimensional scaling plot displayed in Figure 1. Samples recovered from the Atlantic Forest and Tocantins cluster among multiple, divergent groups. Meanwhile TcI from *PARA*_NORTH_, *PARA*_SOUTH_ and Ceara occur among the same or closely related clusters. Remaining clusters represent intermediates between these two extremes. In summary, genetic diversity among some populations looks considerably more fragmented than among others. Mean pair-wise values for D_AS_ and their associated SD seem to reflect this (Table 1).

Given the intense degree of admixture and substructure in several populations we decided not to calculate population specific linkage disequilibrium indices. Substructure is known to inflate such measures and increase the likelihood of a type 1 error [39]. Instead we chose to evaluate congruence between nuclear and mitochondrial genome clustering as evidence for rare genetic exchange events. To make such a comparison we incorporated previously published nuclear and mtDNA data into our dataset [28]. Figure 2 shows the resulting trees and the single recombinant we were able to detect mong the 14 clones assayed – 6824, isolated from *Didelphis albiventris* in the Caatinga, possesses a mitochondrial genome of Amazonian origin. The hypothetical direction of the introgression event (recipient and donor) is detailed in the map inset.

The inclusion of nuclear reference microsatellite profiles from throughout the Americas in Figure 2 provides insight into the wider affinities of the Brazilian isolates. Most notably, isolates belonging to cluster 11 in Figure 1 form a homogenous group that cluster basally, well outside global TcI diversity. *GPI* sequences for this group nonetheless confirmed this group as TcI and no affinities with Tcbat were apparent based on the same target (data not shown).

## Discussion

TcI diversity in Brazil is clearly considerable by comparison to that in the rest of South, Central and North America. Figure 2 shows a comparison of isolates evaluated in this study with those analysed previously [27]. Nuclear genetic data (left hand tree) indicate a clade (corresponding to population 11 in Figure 1) that lies outside the known diversity of TcI in the Americas. The presence of a bat trypanosome among this group led us to suspect that this cluster may be Tcbat, a novel DTU with affiliations to TcI originally isolated from chiroptera in Sao Paolo state, but now recognised as more widespread [40,41]. However, sequence comparison of this clade and Tcbat at the *GPI* gene rejected this hypothesis (data not shown). In contrast, all remaining TcI isolates from Brazil fall alongside their congeners, including isolates from Bolivia and Argentina, but distinct from isolates north of the Amazon basin (Venezuela, North and Central America).

The available data suggest that genetic exchange is a fairly common phenomenon among TcI isolates [25,42], which is also capable of genetic recombination in the laboratory [43]. A consistent feature of genetic exchange events is the uniparental inheritance of mtDNA. At a population level, as well as between DTUs, these events lead to clear instances of mitochondrial introgression [25]. Thus a pair of isolates maybe highly genetically similar on a nuclear level, but lack any affinity between mitochondrial genomes. We identified one such hybrid among those clones we assayed - 6824. In a recent review, it was proposed that ‘different evolutionary pressures and molecular clocks’ between non-coding nuclear microsatellite and coding mtDNA, rather than genetic exchange, might account for such signals of introgression [44]. However, such a theory requires a situation in which two (or more) near identical nuclear genotypes (e.g. 6824 and 9667) experience radically different evolutionary pressures on their mitochondrial genomes, which end up closely resembling the mitochondrial genotype of nearby or sympatric clones, in this case from the same host (*Didelphis albiventris*). Given that this pattern of introgression fits precisely with that observed in hybrids in the laboratory [43], and between DTUs in the field (TcI/TcIV) [45], recombination is the only reasonable explanation.

Of particular interest in our study was the distribution and structure of genetic diversity within and between ecoregions. Admixture was most common in the Atlantic forest region, and largely absent from the Amazon region in Pará state (Figure 1). As such, samples from the Atlantic Forest region have strong affinity with those from around Brazil and are thus distributed across multiple genetic clusters in Figure 1. The inset in Figure 1 provides fine details of parasite genetic diversity in the Atlantic Forest region. Isolates at the northern extreme of this region have predictable affinity with samples from the Caatinga (cluster 5). However, admixture into Atlantic forest from other populations is far less predictable, especially from Amazonia, and the Pantanal, which lie thousands of kilometres from the Atlantic forest. The impact of Atlantic forest fragmentation on species abundance and diversity is well documented (e.g. [46-48]). Most studies report loss of alpha diversity correlating inversely with forest fragment size, within as well as between species [46,49]. In contrast, allelic richness indices in our study suggested substantial *T. cruzi* genetic diversity within the Atlantic Forest (Table 1). However, invasive species introductions are common in the Atlantic Forest region (e.g. [50]), and it seems that several long range introductions from distant populations may also explain the high genetic diversity of TcI in the region. Thus, unlike TcI populations from Amazonia and Caatinga, which generally exhibit high genetic diversity but little admixture, high genetic diversity in the Atlantic Forest region is explained by these introductions and associated admixture. Long-range sylvatic dispersal of *T. cruzi* can be achieved by bats. Indeed, the presence of *T. cruzi* clade trypanosomes in Africa can be explained by rapid aerial dispersal [51]. Cluster 11 contains several isolates from bats, which could explain the geographic diversity of isolates in this clade (Atlantic Forest, Pantanal, Cerrado), as well as its genetic homogeneity. However, other geographically diverse isolate groupings containing Atlantic Forest isolates have no link to volant mammals.

There is a circumstantial link between Atlantic Forest loss (88% of its former extent [52]), human population density, and TcI genetic admixture in the region. *T. cruzi* infection is commonly termed a ‘zoonosis’, which implies unidirectional dispersion from sylvatic transmission cycles to man. Until the successful triatomine eradication campaigns of the 1970s and 1980s, domestic *T. cruzi* infection was endemic throughout much (although not all) of the Atlantic Forest region [21]. It is thus possible that many of these long-range introductions into the Atlantic are ‘enzooses’, i.e. TcI strains imported via immigrant human populations, which subsequently escaped in the local sylvatic environment.

## Conclusions

Rather like primary rainforest, ‘pristine’ sylvatic *T. cruzi* diversity may be now relatively rare in South America, especially where human population densities and infections rates have been historically high. The presence of disturbed and admixed sylvatic *T. cruzi* populations in populous areas has major implications for the effective source attribution and thus future prevention of oral outbreaks [5]. Many such outbreaks have occurred in Brazil in recent years [53]. As such, the discrimination of the source of oral outbreak strains as being from either the local wild population or from another region via the importation of foodstuffs becomes complex. This is because the local wild strains themselves may represent long-range introductions. Nonetheless, admixture among sylvatic parasite populations has a possible role as a proxy for environmental disturbance. Future approaches could involve high-resolution genotyping and focused sampling of Atlantic forest fragments, including co-variates like mammalian and insect biodiversity, to further explore the use of *T. cruzi* as a sentinel species for ecosystem health.

## Competing interests

The authors declare they have no competing interests. The funders had no role in study design, data collection and analysis, decision to publish, or preparation of the manuscript.

## Authors’ contributions

VL and ML generated the data. VL, ML and LM analysed the data. VL, MM and ML wrote manuscript. AJ and ML designed the study. All authors read and approved the final version of the manuscript.

## Supplementary Material

Additional file 1: Table S1 *Trypanosoma cruzi* I isolates evaluated in this study. Click here for file
