# Supplementary material for: Wild Trypanosoma cruzi I genetic diversity in Brazil suggests admixture and disturbance in parasite populations from the Atlantic Forest region
Source: Parasit Vectors. 2014 Jun 5;7:263. doi: 10.1186/1756-3305-7-263 (PMC4062772; doi:10.1186/1756-3305-7-263)
Supplement: Additional file 1: Table S1 — Trypanosoma cruzi I isolates evaluated in this study. [file 1756-3305-7-263-S1.docx]

**Table 1 – *Trypanosoma cruzi* I isolates evaluated in this study**

| **Sample code** | ***Source*** | **Biome** | **Municipality/State** | **Latitude** | **Longitude** | **Genetic cluster based on DAPC** | *A priori* population |
| --- | --- | --- | --- | --- | --- | --- | --- |
| 3510 | *Didelphis albiventris* | Caatinga | Jaguaruana/Ceará | -4.8308 | -37.7814 | 10 | Ceara |
| 6809 | *Rattus rattus* | Caatinga | Jaguaruana/Ceará | -4.8308 | -37.7814 | 7 | Ceara |
| 6812 | *Didelphis albiventris* | Caatinga | Jaguaruana/Ceará | -4.8308 | -37.7814 | 10 | Ceara |
| 6813 | *Didelphis albiventris* | Caatinga | Jaguaruana/Ceará | -4.8308 | -37.7814 | 10 | Ceara |
| 6824* | *Didelphis albiventris* | Caatinga | Jaguaruana/Ceará | -4.8308 | -37.7814 | 10 | Ceara |
| 8622 | *Didephis albiventris* | Caatinga | Jaguaruana/Ceará | -4.8308 | -37.7814 | 7 | Ceara |
| 8648 | *Didelphis albiventris* | Caatinga | Jaguaruana/Ceará | -4.8308 | -37.7814 | 10 | Ceara |
| 9529 | *Rattus rattus* | Caatinga | Jaguaruana/Ceará | -4.8308 | -37.7814 | 7 | Ceara |
| 9531 | *Rattus rattus* | Caatinga | Jaguaruana/Ceará | -4.8308 | -37.7814 | 7 | Ceara |
| 9538 | *Rattus rattus* | Caatinga | Jaguaruana/Ceará | -4.8308 | -37.7814 | 7 | Ceara |
| 9667* | *Monodelphis domestica* | Caatinga | Redenção/Ceará | -4.2261 | -38.7311 | 10 | Ceara |
| 11629 | *Didephis albiventris* | Caatinga | Russas/Ceará | -4.9392 | -37.9786 | 6 | Ceara |
| 11639 | *Didelphis albiventris* | Caatinga | Russas/Ceará | -4.9392 | -37.9786 | 10 | Ceara |
| 11640 | *Didelphis albiventris* | Caatinga | Russas/Ceará | -4.9392 | -37.9786 | 10 | Ceara |
| 8552 | *Didephis albiventris* | Cerrado | Aporé/Goiás | -18.9489 | -51.9086 | 1 | Goiais |
| 9148 | *Gracilinanus sp* | Cerrado | Aporé/Goiás | -18.9489 | -51.9086 | 1 | Goiais |
| 9149 | *Didephis albiventris* | Cerrado | Aporé/Goiás | -18.9489 | -51.9086 | 1 | Goiais |
| 9425* | *Didephis albiventris* | Cerrado | Aporé/Goiás | -18.9489 | -51.9086 | 1 | Goiais |
| 10268 | *Proechimys sp* | Amazon | Cachoeira do Arari/Pará | -1.0044 | -48.9572 | 8 | North Para |
| 10272 | *Didelphis marsupialis* | Amazon | Cachoeira do Arari/Pará | -1.0044 | -48.9572 | 8 | North Para |
| 10285 | *Didelphis marsupialis* | Amazon | Cachoeira do Arari/Pará | -1.0044 | -48.9572 | 8 | North Para |
| 10288 | *Oecomys sp.* | Amazon | Cachoeira do Arari/Pará | -1.0044 | -48.9572 | 8 | North Para |
| 10289 | *Unknown* | Amazon | Cachoeira do Arari/Pará | -1.0044 | -48.9572 | 8 | North Para |
| 10290 | *Didelphis marsupialis* | Amazon | Cachoeira do Arari/Pará | -1.0044 | -48.9572 | 8 | North Para |
| 11604 | *Marmosops murina* | Amazon | Abaetetuba/Pará | -1.7297 | -48.8719 | 4 | North Para |
| 11605 | *Philander opossum* | Amazon | Abaetetuba/Pará | -1.7297 | -48.8719 | 4 | North Para |
| 11609 | *Philander opossum* | Amazon | Abaetetuba/Pará | -1.7297 | -48.8719 | 4 | North Para |
| 11611 | *Philander opossum* | Amazon | Abaetetuba/Pará | -1.7297 | -48.8719 | 8 | North Para |
| 12624* | *Philander opossum* | Amazon | Abaetetuba/Pará | -1.7297 | -48.8719 | 6 | North Para |
| 12625 | *Didelphis marsupialis* | Amazon | Abaetetuba/Pará | -1.7297 | -48.8719 | 8 | North Para |
| 12626 | *Micoureus demerarae* | Amazon | Abaetetuba/Pará | -1.7297 | -48.8719 | 32 | North Para |
| 12628 | *Didelphis marsupialis* | Amazon | Abaetetuba/Pará | -1.7297 | -48.8719 | 6 | North Para |
| 12630* | *Philander opossum* | Amazon | Abaetetuba/Pará | -1.7297 | -48.8719 | 4 | North Para |
| 12631 | *Philander opossum* | Amazon | Abaetetuba/Pará | -1.7297 | -48.8719 | 4 | North Para |
| 12640 | *Didelphis marsupialis* | Amazon | Abaetetuba/Pará | -1.7297 | -48.8719 | 8 | North Para |
| 12667 | *Didelphis marsupialis* | Amazon | Curralinho/Pará | -0.5364 | -49.1842 | 8 | North Para |
| 12668 | *Didelphis marsupialis* | Amazon | Curralinho/Pará | -0.5364 | -49.1842 | 8 | North Para |
| 12964 | *Didelphis marsupialis* | Amazon | Curralinho/Pará | -0.5364 | -49.1842 | 8 | North Para |
| FNS258 | *Canis familiaris* | Amazon | Abaetetuba/Pará | -1.7297 | -48.8719 | 6 | North Para |
| LBT1812 | *Rhodnius pictipes* | Amazon | Belém/Pará | -1.3789 | -48.4756 | 8 | North Para |
| LBT1813 | *Rhodnius pictipes* | Amazon | Belém/Pará | -1.3789 | -48.4756 | 8 | North Para |
| LBT918 | *Canis familiaris* | Amazon | Abaetetuba/Pará | -1.7297 | -48.8719 | 6 | North Para |
| LBT964 | *Rhodnius pictipes* | Amazon | Abaetetuba/Pará | -1.7297 | -48.8719 | 4 | North Para |
| LBT966 | *Rhodnius pictipes* | Amazon | Abaetetuba/Pará | -1.7297 | -48.8719 | 4 | North Para |
| LBT967 | *Rhodnius pictipes* | Amazon | Abaetetuba/Pará | -1.7297 | -48.8719 | 8 | North Para |
| LBT969 | *Rhodnius pictipes* | Amazon | Abaetetuba/Pará | -1.7297 | -48.8719 | 8 | North Para |
| 5324 | *Oecomys sp* | Pantanal | Aquidauana/Mato Grosso do Sul | -19.6806 | -57.3378 | 2 | Pantanal |
| 5340 | *Oecomys sp* | Pantanal | Aquidauana/Mato Grosso do Sul | -19.6806 | -57.3378 | 2 | Pantanal |
| 5355 | *Monodelphis domestica* | Pantanal | Aquidauana/Mato Grosso do Sul | -19.1393 | -56.7958 | 2 | Pantanal |
| FRN46* | *Oecomys sp* | Pantanal | Aquidauana/Mato Grosso do Sul | -19.6806 | -57.3378 | 11 | Pantanal |
| 5666 | *Gracilinanus agilis* | Pantanal | Corumbá/Mato Grosso do Sul | -19.1393 | -56.7958 | 2 | Pantanal |
| 5667 | *Gracilinanus agilis* | Pantanal | Corumbá/Mato Grosso do Sul | -19.1393 | -56.7958 | 2 | Pantanal |
| 5674 | *Monodelphis domestica* | Pantanal | Corumbá/Mato Grosso do Sul | -19.0097 | -57.6547 | 9 | Pantanal |
| 5679 | *Thylamys macrurus* | Pantanal | Corumbá/Mato Grosso do Sul | -19.1393 | -56.7958 | 2 | Pantanal |
| 5698 | *Gracilinanus sp* | Pantanal | Corumbá/Mato Grosso do Sul | -19.1393 | -56.7958 | 2 | Pantanal |
| 7587 | *Gracilinanus agilis* | Pantanal | Corumbá/Mato Grosso do Sul | -19.0097 | -57.6547 | 2 | Pantanal |
| GM288 | *Nasua nasua* | Pantanal | Corumbá/Mato Grosso do Sul | -19.0097 | -57.6547 | 2 | Pantanal |
| GM295 | *Nasua nasua* | Pantanal | Corumbá/Mato Grosso do Sul | -19.0097 | -57.6547 | 2 | Pantanal |
| THY01 | *Thylamys macrurus* | Pantanal | Corumbá/Mato Grosso do Sul | -18.9919 | -56.6313 | 2 | Pantanal |
| 4250 | *Thrichomys apereoides* | Caatinga | São Raimundo Nonato/Piauí | -9.0053 | -45.7114 | 5 | Piaui |
| 4262 | *Thrichomys apereoides* | Caatinga | São Raimundo Nonato/Piauí | -8.4167 | -42.3331 | 5 | Piaui |
| 6183 | *Didelphis albiventris* | Caatinga | São Raimundo Nonato/Piauí | -9.0053 | -45.7114 | 10 | Piaui |
| FNS1* | *Triatoma brasiliensis* | Caatinga | João Costa/Piauí | -8.5103 | -42.4200 | 5 | Piaui |
| M1 | *Didelphis albiventris* | Caatinga | Coronel José Dias/Piauí | -8.8250 | -42.5064 | 10 | Piaui |
| M3 | *Didelphis albiventris* | Caatinga | Coronel José Dias/Piauí | -8.8250 | -42.5064 | 7 | Piaui |
| 645 | *Didelphis marsupialis* | Atlantic Forest | Teresópolis/Rio de Janeiro | -42.9667 | -42.9667 | 11 | Atlantic Forest |
| 762 | *Didelphis* | Atlantic Forest | Silva Jardim/Rio de Janeiro | -22.4117 | -42.9667 | 9 | Atlantic Forest |
| 5563 | *Nectomys squamipes* | Atlantic Forest | Capitão Andrade/Minas Gerais | -19.0700 | -41.8633 | 3 | Atlantic Forest |
| 5565 | *Didelphis aurita* | Atlantic Forest | Capitão Andrade/Minas Gerais | -19.0700 | -41.8633 | 9 | Atlantic Forest |
| 5574 | *Didelphis aurita* | Atlantic Forest | Capitão Andrade/Minas Gerais | -19.0700 | -41.8633 | 9 | Atlantic Forest |
| BF5 | *Rhodnius prolixus* | Atlantic Forest | Teresópolis/Rio de Janeiro | -42.9667 | -42.9667 | 11 | Atlantic Forest |
| BP4 | *Rhodnius prolixus* | Atlantic Forest | Teresópolis/Rio de Janeiro | -42.9667 | -42.9667 | 11 | Atlantic Forest |
| BPT4 | *Rhodnius prolixus* | Atlantic Forest | Teresópolis/Rio de Janeiro | -42.9667 | -42.9667 | 11 | Atlantic Forest |
| C12 | *Philander frenatus* | Atlantic Forest | Teresópolis/Rio de Janeiro | -42.9667 | -42.9667 | 11 | Atlantic Forest |
| C45 | *Philander frenatus* | Atlantic Forest | Teresópolis/Rio de Janeiro | -42.9667 | -42.9667 | 11 | Atlantic Forest |
| C48 | *Philander frenatus* | Atlantic Forest | Teresópolis/Rio de Janeiro | -42.9667 | -42.9667 | 11 | Atlantic Forest |
| C60* | *Philander frenatus* | Atlantic Forest | Teresópolis/Rio de Janeiro | -42.9667 | -42.9667 | 11 | Atlantic Forest |
| D7 | *Didelphis aurita* | Atlantic Forest | Silva Jardim/Rio de Janeiro | -22.6592 | -42.3831 | 5 | Atlantic Forest |
| D8 | *Didelphis marsupialis* | Atlantic Forest | Silva Jardim/Rio de Janeiro | -22.6592 | -42.3831 | 5 | Atlantic Forest |
| G05 | *Didelphis sp.* | Atlantic Forest | Silva Jardim/Rio de Janeiro | -22.5319 | -42.9897 | 9 | Atlantic Forest |
| G15 | *Didelphis marsupialis* | Atlantic Forest | Silva Jardim/Rio de Janeiro | -22.5319 | -42.9897 | 9 | Atlantic Forest |
| G33 | *Didelphis marsupialis* | Atlantic Forest | Silva Jardim/Rio de Janeiro | -22.5319 | -42.9897 | 5 | Atlantic Forest |
| G41* | *Didelphis marsupialis* | Atlantic Forest | Silva Jardim/Rio de Janeiro | -22.5319 | -42.9897 | 8 | Atlantic Forest |
| MLCD44 | *Leontopithecus chrysomela* | Atlantic Forest | Ilhéus/Bahia | -15.2694 | -39.0666 | 5 | Atlantic Forest |
| MLD291 | *Leontopithecus rosalia* | Atlantic Forest | Silva Jardim/Rio de Janeiro | -22.6592 | -42.3831 | 11 | Atlantic Forest |
| MLD490 | *Leontopithecus rosalia* | Atlantic Forest | Silva Jardim/Rio de Janeiro | -22.6592 | -42.3831 | 11 | Atlantic Forest |
| MLD524 | *Leontopithecus rosalia* | Atlantic Forest | Silva Jardim/Rio de Janeiro | -22.6592 | -42.3831 | 11 | Atlantic Forest |
| MLD600 | *Leontopithecus rosalia* | Atlantic Forest | Silva Jardim/Rio de Janeiro | -22.6592 | -42.3831 | 3 | Atlantic Forest |
| MLD632 | *Leontopithecus rosalia* | Atlantic Forest | Silva Jardim/Rio de Janeiro | -22.6592 | -42.3831 | 11 | Atlantic Forest |
| MLD714 | *Leontopithecus rosalia* | Atlantic Forest | Silva Jardim/Rio de Janeiro | -22.6592 | -42.3831 | 3 | Atlantic Forest |
| MLD776c | *Leontopithecus rosalia* | Atlantic Forest | Silva Jardim/Rio de Janeiro | -22.6592 | -42.3831 | 11 | Atlantic Forest |
| MLD877b* | *Leontopithecus rosalia* | Atlantic Forest | Silva Jardim/Rio de Janeiro | -22.6592 | -42.3831 | 3 | Atlantic Forest |
| 7301 | *Didelphis aurita* | Atlantic Forest | Navegantes/Santa Catarina | -26.8989 | -48.6558 | 2 | Santa Catarina |
| 7313 | *Didelphis aurita* | Atlantic Forest | Navegantes/Santa Catarina | -26.8989 | -48.6558 | 2 | Santa Catarina |
| 7344* | *Olygoryzomys nigripes* | Atlantic Forest | Jaborá/Santa Catarina | -27.1703 | -51.7375 | 2 | Santa Catarina |
| 6716 | *Didelphis marsupialis* | Amazon | Itupiranga/Pará | -5.1733 | -49.3656 | 8 | South Para |
| 6723 | *Didelphis marsupialis* | Amazon | Itupiranga/Pará | -5.1733 | -49.3656 | 9 | South Para |
| 6737 | *Didelphis marsupialis* | Amazon | Itupiranga/Pará | -5.1733 | -49.3656 | 8 | South Para |
| 13100 | *Didelphis marsupialis* | Amazon(cerr) | Augustinópolis/Tocantins | -5.4683 | -47.8894 | 8 | South Para |
| 13103 | *Phyllostomus hastatus* | Amazon(cerr) | Augustinópolis/Tocantins | -5.4683 | -47.8894 | 4 | South Para |
| 10171* | *Didelphis albiventris* | Cerrado/Caa | São Raimundo Nonato/Piauí | -9.9667 | -45.7167 | 10 | Tocantins |
| 12903 | *Gracilinanus sp.* | Cerrado | Dianópolis/Tocantins | -11.6278 | -46.8208 | 5 | Tocantins |
| JFV297 | *Desmodus rotundus* | Cerrado | Arraias/Tocantins | -12.9264 | -46.9350 | 11 | Tocantins |
| JFV306* | *Carolia perspicillata* | Cerrado | Arraias/Tocantins | -12.9264 | -46.9350 | 11 | Tocantins |
| JFV307* | *Phyllostomus albicola* | Cerrado | Arraias/Tocantins | -12.9264 | -46.9350 | 11 | Tocantins |
| JFV313 | *Phyllostomus hastatus* | Cerrado | Arraias/Tocantins | -12.9264 | -46.9350 | 11 | Tocantins |
| T.sord15 | *Triatoma sordida* | Cerrado | Posse/Goiás | -1.7297 | -48.8719 | 10 | Tocantins |

*Biologically cloned and used to derive maxicircle sequences.
